# Multiple Wnt/ß-Catenin Responsive Enhancers Align with the *MYC* Promoter through Long-Range Chromatin Loops

**DOI:** 10.1371/journal.pone.0018966

**Published:** 2011-04-20

**Authors:** Gregory S. Yochum

**Affiliations:** Department of Biochemistry and Molecular Biology, The Pennsylvania State University College of Medicine, Hershey, Pennsylvania, United States of America; Texas A&M University, United States of America

## Abstract

Inappropriate activation of *c-Myc* (*MYC*) gene expression by the Wnt/ß-catenin signaling pathway is required for colorectal carcinogenesis. The elevated MYC levels in colon cancer cells are attributed in part to ß-catenin/TCF4 transcription complexes that are assembled at proximal Wnt/ß-catenin responsive enhancers (WREs). Recent studies suggest that additional WREs that control *MYC* expression reside far upstream of the *MYC* transcription start site. Here, I report the characterization of five novel WREs that localize to a region over 400 kb upstream from *MYC*. These WREs harbor nucleosomes with post-translational histone modifications that demarcate enhancer and gene promoter regions. Using quantitative chromatin conformation capture, I show that the distal WREs are aligned with the *MYC* promoter through large chromatin loops. The chromatin loops are not restricted to colon cancer cells, but are also found in kidney epithelial and lung fibroblast cell lines that lack de-regulated Wnt signaling and nuclear ß-catenin/TCF4 complexes. While each chromatin loop is detected in quiescent cells, the positioning of three of the five distal enhancers with the *MYC* promoter is induced by serum mitogens. These findings suggest that the architecture of the *MYC* promoter is comprised of distal elements that are juxtaposed through large chromatin loops and that ß-catenin/TCF4 complexes utilize this conformation to activate *MYC* expression in colon cancer cells.

## Introduction

The c-Myc transcription factor (MYC) is a critical regulator of cellular growth, proliferation and apoptosis [1]. MYC is a member of the basic-helix-loop-helix zipper (bHLHZ) family of transcription factors and dimerizes with the bHLHZ factor MAX to bind DNA. MYC:MAX heterodimers activate gene expression by recruiting protein complexes that contain histone acetyltransferases and chromatin remodelers [2]. When bound to MYC-interacting zinc finger 1 (MIZ-1), MYC represses transcription of target genes [3]. Genome-wide profiling studies have indicated that expression of thousands of genes may be regulated directly by MYC [4]. The importance of maintaining appropriate MYC levels for cellular homeostasis is underscored by the finding that the *MYC* gene is frequently over-expressed in numerous cancers including up to 80% of colon carcinomas [5], [6].

The Wnt/ß-catenin signaling pathway controls cell fate decisions and tissue architecture in the gastrointestinal (GI) tract [7]. Mutations in genes comprising this pathway, most commonly in the adenomatous polyposis coli (*APC*) gene, are found in most cases of colon cancer [8]. Mutant APC protein leads to the inappropriate accumulation of ß-catenin transcription co-activator in the nucleus [9], [10]. Nuclear ß-catenin binds members of the T-cell factor/lymphoid enhancer factor (TCF/Lef) family of sequence specific transcription factors to induce target gene expression [11]. ß-catenin recruits chromatin remodeling and histone modifying complexes that render the chromatin at target genes permissive to transcription [11], [12]. One critical and direct target of nuclear ß-catenin/TCF4 complexes is *MYC* [13]. ß-Catenin/TCF4 complexes were shown to regulate *MYC* expression in colon cancer cells through two proximal Wnt/ß-catenin responsive enhancers (WREs) [13], [14]. The first WRE localizes to a region 0.5 kb upstream from the *MYC* transcription start site (TSS) and was identified using a screen for genes whose expression was negatively regulated by *APC* [13]. The second WRE localized to a region 1.6 kb downstream from the *MYC* transcription stop site and was identified using a functional genomics screen for ß-catenin binding sites [14], [15]. ß-Catenin/TCF4 complexes assembled at the downstream WRE coordinated a chromatin loop with complexes assembled at the promoter proximal 5′ WRE to activate *MYC* expression in response to serum mitogens [16]. These studies highlight the intricate mechanisms that underlie Wnt/ß-catenin dependent activation of *MYC* gene expression in colon cancer cells.

A careful analysis of data generated by several genome-wide association studies led to the identification of another WRE that regulates *MYC* expression. An approximately 500 kb span of chromosome 8q24 is a hot spot for single nucleotide polymorphisms (SNPs) that confer risk to colon, prostate and breast cancers [17], [18], [19]. This span is depleted of protein-coding genes so the molecular mechanisms that linked the SNPs to disease risk were elusive. Analysis of one SNP, rs6983267, has begun to shed light on this conundrum. DNA sequence analysis found that the colon and prostate cancer-associated rs6983267 SNP maps to a canonical TCF4 motif [20], [21]. The risk-associated allele conferred stronger TCF4 binding and enhanced Wnt activity in heterologous reporter assays when compared to the non-risk allele [21], [22]. Using chromatin conformation capture (3C), numerous groups reported that the rs6983267 WRE was positioned near the proximal *MYC* promoter [20], [22], [23], [24]. Together these reports offered one explanation for how rs6983267 may confer risk to colon cancer and importantly demonstrated that enhancers that reside as far as 0.35 Mb away from *MYC* can influence *MYC* expression levels.

Two recent studies have expanded upon the idea that the gene desert on chromosome 8q24 harbors additional enhancer elements and that these enhancers may loop to distal target genes. Jia et. al. profiled a 5 Mb segment of chromosome 8 for RNA expression, histone modifications and RNA polymerase two (RNAP) binding [25]. They found that although elements contained nucleosomes with histone modifications known to demarcate enhancers, most transcripts that initiated from this region were expressed at very low levels [25]. *MYC* gene expression however, was readily detected [25]. Using 3C, Ahmadiyeh et. al. found that enhancers that localized to the risk regions within 8q24 formed tissue specific and long-range interactions with *MYC* [23]. In addition they showed that interactions between the risk regions and neighboring elements throughout 8q24 also occurred through chromatin loops albeit at a lower frequency than interactions with *MYC*. These findings suggest that the gene desert region of chromosome 8 is prone to forming chromatin loops and that additional enhancer elements within this region might align with the *MYC* promoter through long-range interactions.

In the present study, I characterize five novel ß-catenin/TCF4 binding sites that were identified using a ChIP-Seq screen for ß-catenin binding regions in the human HCT116 colon cancer cell line [26]. These binding sites localize to a 50 kb segment between regions 128,296,391 and 128,347,610 on chromosome 8 that is over 400 kb upstream from the *MYC* transcription start site. I show that nucleosomes that co-localize with the ß-catenin/TCF4 binding sites contain histones that are decorated with modifications on their amino-terminal tails that are known to demarcate both enhancer and promoter elements. Using chromatin conformation capture (3C), I find that these novel WREs are positioned next to the *MYC* promoter through large chromatin loops. Surprisingly, these loops are not restricted to colon cancer cells, but are also found in cells that lack ß-catenin/TCF4 chromatin associated complexes. The interactions between three of the five distal enhancers with the *MYC* promoter were induced by serum mitogens. Together these data suggest that long-range chromatin loops position distal enhancer elements to the *MYC* promoter and that this conformation is poised to recruit ß-catenin/TCF4 complexes to regulate *MYC* expression in colon cancer cells.

## Methods

### Cell culture

HCT116 human colon cancer cells (ATCC number CCL-247) and HEK293 cells were cultured as previously described [14]. TIG-1 fibroblasts (Coriell Cell Repositories, number AG06173) were cultured in Modified Eagle Medium (Invitrogen) supplemented with 10% fetal bovine serum, 100 units/ml penicillin, 100 units/ml streptomycin, and 5 mM L-glutamine. Cells were maintained at 37°C and 5% CO_2_.

### Chromatin Immunoprecipitation (ChIP)

Antibodies used in ChIP assays included: 3 µg of anti-ß-catenin (BD Transduction, 610154), 3 µg of anti-TCF4 (Millipore, 05-511), 5 µl of anti-RNAP (Covance, 8WG16), 3 µg of anti-TBP (Abcam, ab818-100), 3 µg of anti-RNAPS5 (Abcam, ab817-100), 5 µl of anti-H3K4me3 (Millipore, 07-473), 5 µl of anti-H3K4me 1 (Abcam, ab8895-50), 3 µg of anti-H3K9,K14 Ac (Millipore, 06-599) and 2.5 µl of anti-PanCT H3 (Millipore, 06-866). ChIP assays contained 1×10^7^ cells and were conducted as reported with some modifications [26], [27]. Formaldehyde-fixed cells were collected in ChIP lysis buffer (150 mM NaCl, 5 mM EDTA, pH = 8, 1% Triton X-100, 0.5% NP40, and 50 mM Tris-HCl, pH = 7.5) and the chromatin was sheared to an average size of ∼600–800 bp using a Misonix Ultrasonic XL-2000 Liquid Processor (5×20 s, output wattage 7, with 45 s rest intervals on ice between pulses). Sonicated chromatin was clarified and precipitated as described [26], [27]. Primers were designed using Primer3 software to an 800 bp DNA segment that centered on the ß-catenin ChIP-Seq peak region. The sequences of the primers used are listed in Table S1. Where indicated, real time PCR data is represented as fold levels over control where the control is an average of the signal generated using two distinct primer sets that anneal to regions flanking *MYC* [14].

### Sequential ChIP

The sequential ChIP assays were conducted as described in Shang et. al. with some modifications [28]. Formaldehyde-fixed and sonicated chromatin was precipitated overnight with 5 µl of anti-H3K4me3 antibodies. Captured immunocomplexes were re-suspended in 100 µl of 10 mM DTT and incubated for 1 hour at 37°C on a rotating platform. Eluted complexes were diluted 50 fold in ChIP lysis buffer and precipitated with indicated antibodies overnight at 4°C on a rotating platform. The DNA fragments were purified and assessed by real-time PCR.

### ChIP-Seq

The ß-catenin ChIP-Seq screen in HCT116 cells is described in Bottomly et al. [26].

### Quantitative reverse transcription-PCR

RNA extraction, cDNA synthesis and real-time PCR were conducted as described [26].

### Western blot analysis

HEK293 or HCT116 cells were suspended in 0.5 ml of Radio-Immunoprecipitation Assay (RIPA) buffer (Thermo Scientific, 89901) containing protease inhibitors (Roche complete, 11 697 498 001). The cells were lysed by brief sonication using a Misonix XL-2000 liquid processor (2×5 s output wattage 4, with 45 s rest intervals on ice between pulses). The lysates were clarified by centrifugation for 10 min at 14,000 rpm and the protein content was quantified using protein assay reagent (Bio-Rad, 500-0006). For each sample, 20 µg of protein was subjected to polyacrylamide gel electrophoresis and the proteins were transferred to a nitrocellulose membrane. Standard western blot methodology was used to visualize proteins. Antibodies used in the analysis included anti-c-Myc 9E10 (Santa Cruz, SC40), 1∶250 dilution; and anti-ß tubulin (Sigma, T5201), 1∶1000 dilution. Band intensities were quantified using ImageJ software (http://rsbweb.nih.gov/ij/).

### Electroporation of HCT116 cells

The dominant negative TCF4 (pcDNAdnTCF4) was obtained from Addgene (kindly deposited by Dr. B. Vogelstein). 10 µg of pcDNAdnTCF4 or a GFP expressing plasmid were electroporated into 5×10^6^ HCT116 cells using an Amaxa nucleofector II device.

### Quantitative chromatin conformation capture (q3C)

Chromatin conformation capture was conducted as described with minor modifications [16], [26]. The cross-linked chromatin was digested overnight with 40 µl (800 U) of Hind III (New England Biolabs) and Hind III was heat-inactivated for 20 min at 65°C prior to ligation [16]. Real-time PCR was conducted as for ChIP except that reactions were cycled at 95°C for 3 min and then 50 cycles of 94°C 10 s, 60°C 20 s, and 72°C for 45 s. Several measures were taken to control for PCR and primer efficiencies. First, the primers that spanned the six Hind III sites interrogated were designed using Primer3 software and the annealing temperatures were set between 59°C and 62°C. In control reactions, each primer set generated a single PCR product of the expected size and each product was sequenced and confirmed to be the correct identity. To establish a reference for the quantitative PCR analysis, plasmids harboring each of the six PCR amplicons were mixed at equivalent molar concentrations, digested with Hind III and all compatible DNA ends were joined with T4 ligase (NEB). The ligated products were then amplified in separate PCR reactions with the downstream anchor primer LL2 and upstream primers AL1, BL1, CL1, DL1, and EL1 (see Table S1 for primer sequences). The single PCR products generated in these reactions were subcloned into the TOPO TA vector and sequenced. Independent TOPO plasmids harboring these 3C products generated *in vitro* were then mixed at equal molar concentrations. This mixture was serially diluted and used as a control in the quantitative real-time PCR reactions. The 2 ^ΔCt^ method was used to represent amplified products where 10^−8^ ng of TOPO plasmids harboring control PCR products served as the reference. Control experiments were conducted as described and it was confirmed that all 3C DNA products analyzed in this report required the addition of Hind III and ligase to the reaction [16], [26].

## Results

### Identification of putative Wnt/ß-catenin responsive enhancers on chromosome 8q24

**Figure 1:**
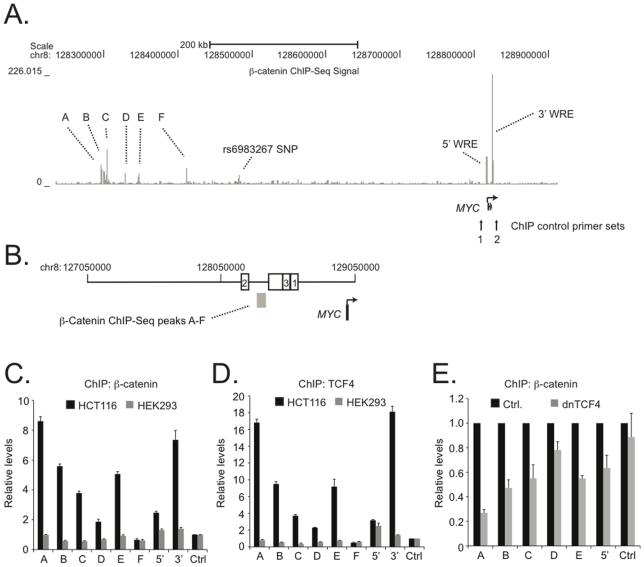
ß-Catenin and TCF4 bind regions on 8q24 that were identified by a ß-catenin ChIP-Seq screen conducted in the HCT116 human colon cancer cell line. (**A**) A diagram of novel ß-catenin binding regions (labeled A through F) and characterized Wnt responsive enhancers (labeled 5′ WRE and 3′ WRE) that were identified in the screen. The gray peaks indicate the sequenced reads collected at each region with the scale of the peak height indicated at the left of the figure. (**B**) Relative positions for the ß-catenin ChIP-Seq peaks on chromosome 8 (gray box). The white boxes indicate risk regions for prostate (1,2,3), colorectal (3), and breast cancers (unlabelled). This diagram was modified from a figure in Jia. et. al [25]. (**C**) Real-time PCR analysis of DNA elements precipitated using ß-catenin specific antibodies in ChIP assays conducted in HCT116 (black bars) or HEK293 (gray bars). A through F corresponds to the regions depicted in Figure 1A with 5′ and 3′ corresponding to the 5′ WRE and 3′ WRE respectively. Control (Ctrl.) is the average signal obtained from two regions in proximity to *MYC* that do not associate with appreciable levels of ß-catenin or TCF4 and data is represented as relative levels of binding over control. The vertical arrows in **A** indicate the positions of the control regions interrogated in the ChIP assays. (**D**) As in **C**, except that TCF4 antibodies were used in the ChIP assays. (**E**) Real-time PCR analysis of ChIP assays conducted with ß-catenin specific antibodies on HCT116 cells that were first transfected with a plasmid expressing dnTCF4 or a control plasmid. Data is represented as levels of ß-catenin binding in dnTCF4 expressing cells relative to control. In **C**, **D** and **E** error is SEM.

We previously conducted a ChIP-Seq screen to identify ß-catenin associated regions in the HCT116 human colorectal cancer cell genome [26]. A computational analysis of the 2,168 high-confidence ß-catenin binding regions identified found that most (95%) were within 495 kb of a protein-coding gene. Under these parameters, many of the ß-catenin target genes in the library contained multiple ß-catenin binding sites. One such gene was the *c-Myc* proto-oncogene (*MYC*). In addition to the well-characterized 5′ and 3′ proximal WREs, at least seven putative binding sites localized to regions far upstream of the *MYC* transcription start site (Figure 1A, Table S1) [13], [14]. These sites were represented by 21 to 70 sequence reads and other targets in the ChIP-seq library that were similarly represented had a high probability of binding ß-catenin in repeat ChIP assays (87.5%) [26]. One of the seven peaks corresponded to the −335 kb WRE that contains SNP rs6983267 and was shown to interact with the *MYC* promoter through a chromatin loop [20], [22], [23], [24]. Others and we have since confirmed ß-catenin and TCF4 binding to the -335 kb WRE and because its function has been studied in colorectal cancer cells, it was not considered further in this report [24], [26]. The six other binding sites were designated A through F and localized near but not within, regions on chromosome 8 that are associated with breast, colon and prostate cancer risk (Figure 1A, B) [25].

The chromosomal span harboring the putative ß-catenin binding regions A-F is referred to as a gene desert due to the paucity of annotated transcripts. Recently, RNAs that emanated from this region were profiled and it was found that *MYC* was the only gene prominently expressed [20], [25]. However, this region contains nucleosomes with histone modifications that demarcate enhancer elements. Therefore in this study, I focused on testing the hypothesis that the six upstream ß-catenin binding regions identified in the ChIP-Seq screen potentially regulated *MYC* expression in *cis* through large chromatin loops.

To determine whether ß-catenin bound regions A through F, I first used chromatin immunoprecipitation assays (ChIP) with ß-catenin specific antibodies. These assays were conducted in HCT116 colorectal cancer cells that contain abundant levels of nuclear ß-catenin and, as a control, in HEK293 cells that contain much reduced levels of nuclear ß-catenin [16], [29]. The ß-catenin bound chromatin isolated by ChIP was assessed using specific oligonucleotides and quantitative real time PCR. ß-Catenin binding to the 5′ and 3′ WREs was readily detected in HCT116 cells as compared to the background binding seen at control regions (Figure 1C, labeled 5′, 3′ and Ctrl). The control regions used localize approximately 5 kb upstream from the *MYC* transcription start site and 5 kb downstream from the *MYC* transcription stop site, respectively (Figure 1A). Only marginal levels of binding to the 5′ and 3′ WREs were seen in HEK293. I then used oligonucleotides designed against regions A–F in real-time PCR reactions and found that like the 5′ and 3′ WREs, ß-catenin bound to elements A-E in HCT116 cells and not HEK293 cells. The levels of binding ranged from two-fold over control (site D) to approximately 8.5-fold over control (site A). Region F failed to bind ß-catenin and it is therefore likely a false positive in the ChIP-Seq library [26]. Nonetheless, this analysis confirmed ß-catenin binding to five regions localizing far upstream of *MYC*.

ß-Catenin recruitment to enhancer elements in colon cancer cells occurs predominantly through interactions with the T-cell factor family member TCF4 [9]. Using TCF4 specific antibodies and ChIP assays, I next determined whether TCF4 co-occupied elements A through E with ß-catenin. TCF4 association with elements A–E was readily detected in HCT116 cells (Figure 1D). TCF4 binding was largely absent in HEK293 cells except at the *MYC* promoter. To determine whether TCF4 was recruiting ß-catenin to these sites, a dominant negative TCF4 plasmid (dnTCF4) was introduced into HCT116 cells and ß-catenin ChIP assays were conducted 24 hours later. TCF4 protein produced from this plasmid lacks the ß-catenin interaction domain and its expression should therefore compete with the formation of functional ß-catenin/TCF4 complexes on elements A through E [13]. Expression of dnTCF4 impaired ß-catenin binding to elements A–E (Figure 1E) while no effect was seen at the control region. Together, these results suggest that TCF4 recruits ß-catenin to regions within the gene desert on chromosome 8q24.

**Figure 2:**
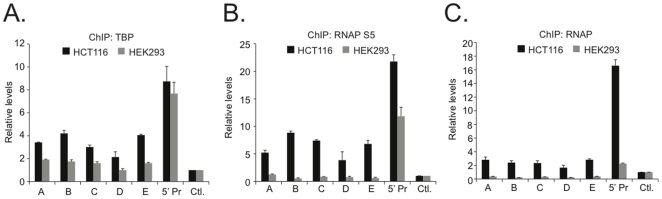
The TATA binding protein (TBP) and RNA Polymerase II (RNAP) associate with the ß-catenin/TCF4 enhancers in HCT116 cells. (**A**) Real-time PCR analysis of ChIP assays conducted in HCT116 (black bars) or HEK293 cells (gray bars) using specific antibodies against TATA binding protein (TBP). Data is represented as fold binding over control (Ctl.). (**B**) As in **A**, except that antibodies specific for RNAP phosphorylated on serine 5 of its carboxy-terminal domain were used in the ChIP assays. (**C**) As in **A** and **B** except antibodies that detect total RNAP were used in the ChIP assays. In each experiment, error bars represent SEM.

One function for transcription factor complexes bound to enhancer elements is to facilitate formation of a pre-initiation complex of general transcription factors [30]. One general transcription factor is the TATA-binding protein (TBP) which plays a role in the initial recognition of core promoter DNA elements [31]. Using ChIP assays, I tested whether TBP associated with elements A through E. While TBP was detected at A through E in HCT116 cells, levels of TBP at these sites were only slightly above control in HEK293 cells (Figure 2A). TBP was found at the *MYC* promoter in both cells as *MYC* is expressed in each cell type (Figure S1). RNAP binding was next interrogated by ChIP. In these assays, I first used antibodies specific for the species of RNAP that is phosphorylated on serine 5 within the carboxy terminal domain (RNAP S5). RNAP S5 is found primarily at the core promoter regions of target genes [32]. As was the case for TBP, RNAP S5 bound enhancers A through E as well as the 5′ promoter in HCT116 cells (Figure 2B). However, RNAP S5 was absent at enhancers A through E in HEK293 cells with significant levels only detected at the promoter region. Similar results were seen using an antibody that recognizes total RNAP (Figure 2C). Together, these results suggest that enhancers A through E recruit functional RNAP transcription complexes in HCT116 cells and not HEK293 cells.

**Figure 3:**
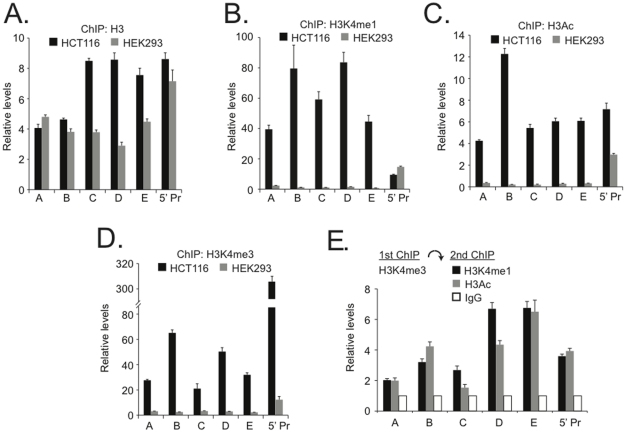
Post-translational histone modifications that characterize enhancer and promoter elements co-localize to distal ß-catenin/TCF4 binding regions. Post-translation modifications to the amino terminal tails of histone H3 (H3) were interrogated using specific antibodies in chromatin immunoprecipitation assays conducted in HCT116 and HEK293 cells. Real-time PCR analysis was used to analyze chromatin that was precipitated with specific antibodies raised against (**A**), H3, (**B**) H3 monomethylated lysine 4 (H3K4me1), (**C**) H3 acetylated on lysines 9 and 14 (H3Ac) and (**D**) H3 trimethylated on lysine 4 (H3K4me3). Black bars indicate results from experiments conducted in HCT116 cells and gray bars indicate those from experiments conducted in HEK293 cells. In **B**, **C**, and **D**, data is normalized to the levels of bulk H3 depicted in A and error is SEM. (**E**) Sequential ChIP in HCT116 colon cancer cells where formaldehyde-fixed and sonicated chromatin was first precipitated with antibodies specific to H3K4me3. The protein-DNA complexes were then eluted from the agarose beads and re-precipitated with antibodies specific to H3K4me1 (black bars), H3Ac (gray bars) or IgG (open bars) as control. Purified DNA was then amplified with oligonucleotides specific to regions A through E and the promoter (5′ Pr) as indicated. Data is normalized to levels of chromatin precipitated with IgG and error bars represent SEM.

Recently, Jia et. al. profiled post-translational histone modifications within nucleosomes throughout the gene desert region of chromosome 8q24 [25]. An analysis of this data and data provided by the ENCODE consortium indicated that nucleosomes with post-translational modifications that demarcate enhancer elements co-localize to the ß-catenin/TCF4 binding sites A through E [25], [33]. Therefore ChIP assays were next conducted to determine whether a subset of histone modifications were present within the nucleosomes at these regions. Because enhancer elements can be depleted of nucleosomes, I first determined whether histone H3 could be detected at ß-catenin binding sites A through E. Histone H3 was detected at each ß-catenin/TCF4 binding site as well as the *MYC* promoter (Figure 3A). For enhancers A, B and the promoter, comparable levels of H3 were present in HEK293 and HCT116 cells whereas approximately two fold higher levels of H3 was found at enhancers C, D, and E in HCT116 cells relative to HEK293 (Figure 3A). I next interrogated the presence of specific modifications to the amino terminal tails of H3 within these nucleosomes. Monomethylated lysine 4 on histone H3 (H3K4me1) has been shown to primarily associate with enhancer elements [34], [35]. Indeed, abundant levels of H3K4me1 co-localized to A through E in HCT116 cells (Figure 3B). In comparison to HCT116 cells, H3K4me1 levels were much reduced at sites A through E in HEK293 cells although this modification was detected at the promoter region (Figure 3B). These results support the conclusion that ß-catenin binding sites A through E demarcate enhancer elements that function in HCT116 cells and not in HEK293 cells.

Next, levels of histone H3 acetylated at lysines 9 and 14 (H3Ac) and tri-methylated at lysine 4 (H3K4me3) were analyzed by ChIP. H3Ac is commonly found at both enhancer and promoters of expressed genes whereas H3K4me3 preferentially localizes to gene promoters [34], [35], [36]. As expected, since *MYC* is transcribed in both HEK293 and HCT116 cells, H3Ac and H3K4me3 were readily detected at the *MYC* promoter (Figure 3C and 3D). Increased levels of each modification were found at the *MYC* promoter in HCT116 cells relative to HEK293 cells. While little H3Ac and H3K4me3 were detected at enhancers A through E in HEK293 cells, these modifications were abundant at these sites in HCT116 cells.

There are many possible scenarios that could explain the co-localization of enhancer and promoter-associated histone modifications at the distal ß-catenin enhancers in HCT116 cells. Each modification may reside on the same *MYC* allele, or two distinct populations of alleles (or cells) may have been interrogated in the ChIP assays. Sequential ChIP assays were conducted to distinguish between these possibilities. First standard ChIP assays were performed to isolate H3K4me3-associated chromatin. The captured complexes were eluted from the H3K4me3 antibody, diluted in ChIP buffer and then re-precipitated with antibodies against H3K4me1 or antibodies against H3Ac. Irrelevant IgG was used as control in the second precipitation to monitor non-specific interactions. Results from sequential ChIP assays clearly indicated that H3K4me3 and H3K4me1 or H3Ac modifications reside on the same allele for each of the distal enhancers A through E (Figure 3E). Interestingly, each modification was present at the *MYC* promoter as well (Figure 3E). Because enhancers A through E reside in a gene desert, it is unlikely that deposition of H3K4me3 histones at these regions is demarcating previously unidentified transcripts. Rather co-enrichment of these modifications may reflect a physical interaction between the distal elements and the *MYC* promoter through large chromatin loops.

### Large chromatin loops align the distal enhancers with the *MYC* promoter

**Figure 4:**
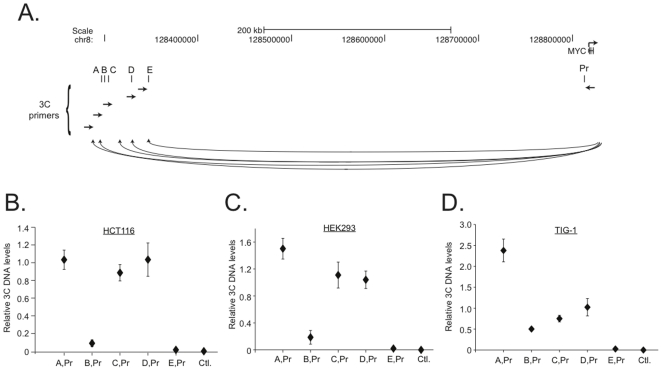
The distal enhancers are juxtaposed to the *MYC* promoter through large chromatin loops. (**A**) Diagram of regions interrogated for chromatin looping using quantitative chromatin conformation capture analysis (q3C). The directed q3C analysis tested whether a Hind III fragment upstream of the *MYC* promoter (the “bait”) interacted with Hind III fragments that are adjacent to each of the upstream enhancers (A, B, C, D, or E). (**B**) Real-time PCR analysis was conducted with primers specific to the regions indicated on the X-axis following 3C reactions. The data is expressed as relative 3C DNA levels using the 2^ΔCt^ method where the reference Ct is obtained from genomic DNA that is spiked with a plasmid harboring the 3C product indicated and the sample Ct is generated from 3C assays conducted in HCT116 (**B**), HEK293 cells (**C**), or TIG-1 fibroblasts (**D**). Ctrl. is a region of the *tubulin* gene that flanks two distal Hind III sites, but does not form a chromatin loop. Error bars represent SEM.

Chromatin conformation capture (3C) is a powerful technique used to identify potential long-range interactions between distal elements [37], [38]. 3C is a PCR-based method where a product is generated only if the DNA elements in question are aligned when cells are fixed with formaldehyde. In most instances, these interactions occur through chromatin loops that are formed between regions on the same chromosome (in *cis*) although interactions between regions on distinct chromosomes (in *trans*) have been described [38]. The potential interactions between the distal *MYC* enhancers and the *MYC* promoter are depicted in Figure 4A. In this experiment, a Hind III fragment at the *MYC* promoter is used as “bait” to test for specific interactions with Hind III fragments adjacent to enhancers A through E (the “preys”). For each PCR reaction, the forward primer anneals upstream of the distal enhancer and the reverse primer, which is common to each reaction, anneals downstream from the Hind III site in the *MYC* promoter. Interactions between the *MYC* promoter and enhancers A, C and D were readily detected in HCT116 cells (Figure 4B). Interactions between the *MYC* promoter and enhancers B and E were somewhat less abundant, but still detectable. As a control, a Hind III flanked region of *tubulin* was tested and this region failed to yield product in 3C assays (Figure 4A, Ctrl). Sequencing the PCR products generated in these reactions confirmed that the *MYC* promoter interacts with all five distal enhancers in HCT116 cells through large chromatin loops.

To determine whether the interactions between the distal enhancers and the *MYC* promoter were dependent on chromatin-associated ß-catenin/TCF4 complexes, 3C assays were next conducted in HEK293 cells. HEK293 cells lack ß-catenin and TCF4 binding to enhancers A through E (Figure 1B and C). Therefore, one would expect a decrease in the abundance of 3C products generated in this cell line if the distal/promoter interactions required ß-catenin/TCF4. Surprisingly, the chromatin loops were detected in HEK293 cells and at most sites the levels of 3C products paralleled the levels detected in HCT116 cells (Figure 4C). I then tested for interactions between the distal enhancers and the *MYC* promoter in the non-transformed TIG-1 fetal human fibroblast cell line [14], [39]. Like HEK293 cells, TIG-1 fibroblasts lack chromatin associated ß-catenin/TCF4 complexes [14], [39]. Results depicted in Figure 4D demonstrate that alignments between all five distal enhancers and the *MYC* promoter were detected in TIG-1 cells. This analysis clearly indicates that the chromatin loops between distal enhancers and the *MYC* promoter are not restricted to colon cancer cells.

**Figure 5:**
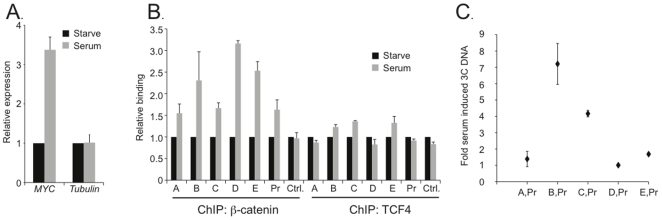
Serum mitogens induce the positioning of a subset of the distal enhancers to the *MYC* promoter. (**A**) HCT116 cells were grown to confluence, deprived of serum for 48 hrs and then stimulated with serum for 2 hours. Real-time PCR analysis was used to quantify *MYC* or *Tubulin* expression levels in cDNAs synthesized from RNAs isolated from starved (black bars) or serum-induced (grey bars). (**B**) Real-time PCR analysis of DNA fragments precipitated with either ß-catenin or TCF4 antibodies in ChIP assays conducted from HCT116 cells treated as in **A**. (**C**) Quantitative 3C analysis detected interactions between enhancers A through E and the *MYC* promoter in serum starved and serum activated HCT116 cells. In **A**, **B** and **C** data is normalized to levels measured in starved cells and error bars represent SEM.

I hypothesized that a ubiquitous signal transduction pathway might regulate the frequency of the chromatin loops. *MYC* expression increases in numerous cell types in response to serum mitogens [40]. To test whether exposure to serum mitogens might regulate formation of the chromatin loops, HCT116 cells were grown to a confluent monolayer and cultured in serum-depleted medium for 48 hours. This treatment is sufficient to cause these cells to exit the cell cycle and enter quiescence [14], [41]. The cells were then exposed to serum for 2 hours. In line with our previous reports, *MYC* expression is induced by serum in HCT116 cells (Figure 5A) [14]. ß-Catenin and TCF4 binding to the distal enhancers and *MYC* promoter region was next interrogated as cells were exposed to serum. We have previously shown that ß-catenin binding to the *MYC* promoter is induced by serum and results presented here confirm that finding (Figure 5B, Pr) [14]. In addition, ß-catenin binding to enhancers A–E was induced by serum whereas TCF4 binding to enhancers B, C, and E was only slightly induced. Finally, serum-induced chromatin looping was tested by 3C. While the chromatin loops between A and D and the *MYC* promoter were insensitive to serum treatment, the interactions between B, C, and E with the *MYC* promoter were increased by serum (Figure 5C). Interestingly, all five chromatin loops were present in quiescent cells. Together, these results suggest that both static and dynamic loops mediate the alignment of the distal *MYC* enhancers and the *MYC* promoter in colon cancer cells.

## Discussion

To understand Wnt/ß-catenin-dependent regulation of target gene expression in colon cancer cells, we previously mapped ß-catenin binding sites throughout the genome of HCT116 human colon cancer cells using the ChIP-Seq methodology [26]. ChIP assays rely on the use of a cross-linking agent that indiscriminately traps all protein-protein and protein-DNA interactions at a given time. Thus transcription factor binding regions uncovered in such screens do not necessarily demarcate enhancer elements [42]. One challenge to the field of functional genomics is to separate productive from non-productive binding events. In this report, I have used information gleaned from our previous ß-catenin ChIP-Seq library to characterize five novel ß-catenin/TCF4 sites that localize to a gene desert on chromosome 8q24. Several lines of evidence support the conclusion that these binding sites demarcate enhancer elements in human colon cancer cells. First, ß-catenin, TCF4, TBP and RNAP bind these sites *in vivo*. Second, nucleosomes that localize to the sites contain histones H3K4me1, H3Ac and H3K4me3. Third, these elements are positioned adjacent to the *MYC* promoter through large chromatin loops and in some cases these loops are induced by serum mitogens. Thus, five novel WREs bind functional ß-catenin/TCF4 complexes and interact with the *MYC* gene locus.

The data presented here indicate that a series of chromatin loops are juxtaposed to the *MYC* promoter at levels equivalent in colon carcinoma and non-colon carcinoma cell lines. However, permissive chromatin marks are deposited at the distal enhancers in colon cancer cells, only. This observation supports a model whereby the constitutive ß-catenin/TCF4 nuclear complexes found in colon cancer cells utilize a pre-existing promoter/distal enhancer configuration to activate *MYC* expression. These distal enhancers are not utilized in HEK293 even though they are juxtaposed to *MYC*. This model offers one explanation for why colon cancers often have increased *MYC* expression relative to non-colon cancer cells. Indeed, HCT116 colon cancer cells have approximately three-fold higher levels of *MYC* transcript and two-fold higher levels of MYC protein when compared to levels detected in HEK293 kidney cells (Figure S1). These findings indicate that although distal elements may be positioned near gene promoter regions, they do not necessarily function as classical enhancers.

It is interesting to note that the gene desert region of chromosome 8 is a hot spot for cancer associated SNPs and harbors numerous enhancer elements that form long-range interactions with the *MYC* gene [23]. This region does in fact contain evidence for transcriptional activity [23], [25]. While the functions of the non-coding transcripts in this region are not fully characterized, perhaps it is the process of transcription that maintains an open chromatin structure to facilitate chromatin looping. Unlike the −335 kb WRE that is associated with SNP rs6983267, the five WREs described in this report are not contained within the regions associated with cancer risk. However, Ahmadiyeh et. al. have clearly shown using 3C analysis that the colon cancer risk region can physically interact with numerous elements on chromosome 8q24 in addition to the *MYC* promoter [23]. One such interaction overlaps the ß-catenin/TCF4 enhancer elements A through E. It is therefore possible that through ternary interactions, multiple enhancers are concurrently juxtaposed to *MYC*. However, current 3C methodology cannot distinguish between concurrent interactions at a single allele or independent interactions at multiple alleles.

In a prior study, we characterized the association of a downstream WRE with the *MYC* promoter through a chromatin loop [16]. That interaction was absent in quiescent cells, but transient interactions were stimulated by serum mitogens. Interestingly, the long-range chromatin loops described in the current study were present in quiescent HCT116 cells (Figure 5C). Because *MYC* is transcriptionally repressed during quiescence, this finding suggests that gene activity is not required for chromatin looping. Interestingly, interactions between enhancers B, C and E with the *MYC* promoter were induced by serum mitogens suggesting that a subset of long-range interactions participate in growth factor stimulation of *MYC* expression. Identifying the signaling pathways that control the positioning of distal enhancers at the *MYC* locus is an area of active investigation in my lab.

ß-Catenin is primarily recruited to chromatin in colonocytes through interactions with TCF4 [9]. Therefore, I searched the enhancer elements for the presence of a consensus TCF4 motif (T/A-T/A-C-A-A-A-G or C-T-T-T-G-A/T-A/T) or the evolutionarily conserved TCF4 motif (A-C/G-T/A-T-C-A-A-A-G or C-T-T-T-G-A/T-C/G-T) [26], [43]. Each enhancer contained at least one consensus motif (Table S1). In addition to consensus TCF4 motifs, the five WREs described here and the 3′ WRE described in our previous reports [14], [16] contain one or more consensus DNA motif known to bind AP-1. AP-1 is a dimeric transcription factor comprised of members of the Jun and Fos families [44]. Growth factors stimulate AP-1 activity in the nucleus through phosphorylation dependent recruitment of chromatin modifying complexes including CBP and p300 [44], [45]. The presence of TCF4 and AP-1 motifs could indicate that an interplay between ß-catenin/TCF4 complexes and AP-1 factors might specify a subset of mitogen responsive WREs that control *MYC* expression. These observations could suggest that these six WREs might have redundant functions in mitogen and Wnt/ß-catenin activation of *MYC* expression. However, our current analysis is limited to HCT116 cells that are derived from a late stage colorectal carcinoma. We have initiated studies in a mouse model system to begin to decipher the roles of Wnt/ß-catenin and mitogen regulation of *MYC* expression in normal gastrointestinal physiology and in the pathophysiology of colon cancer.

Several reports suggest that transcription factors assembled at distal enhancers control the chromatin architecture at target promoter regions [46]. Our previous work implicated a role for ß-catenin/TCF4 complexes in mediating the interaction between the *MYC* 3′ WRE and the *MYC* promoter in colon cancer cells [16]. Because positioning of the distal elements A-E with the MYC promoter was detected in non-colon cancer cells lines that lack chromatin associated ß-catenin/TCF4 complexes, it is unlikely that these factors are required to form the chromatin loops. Recent studies have pointed to the role of cohesin in mediating chromatin loops between distal elements and target genes in *cis* [47], [48]. The well-established role for cohesin is to hold sister chromatids together to ensure proper chromosome segregation during mitosis [49], however an important role for cohesin in regulating gene expression by linking distal enhancers with proximal promoters has been demonstrated [50]. Importantly, this interaction was facilitated by the mediator complex which plays a critical and global role in connecting transcriptional co-activator complexes with general transcription factors and the RNAP holoenzyme [30]. Thus it is possible that the mediator/cohesin complex will also serve important roles in mediating interactions between distal WREs and the *MYC* gene.

*MYC* expression is tightly regulated at the transcriptional level. Elegant work from the Evan laboratory has demonstrated that the biological and tissue specific responses to MYC levels are dose-dependent [51]. Slight increases in the level of MYC can favor hyperplasia and ultimately carcinogenesis. MYC plays a critical role in colon carcinomas that arise from de-regulated Wnt/ß-catenin signaling [52]. The interplay between the precise mechanisms that lead to oncogenic MYC levels in colon cancer cells is yet to be fully understood. While numerous enhancer elements are positioned adjacent to *MYC* proximal promoter, it will be very difficult to functionally ascertain the contribution of each enhancer element to the transcriptional output from the *MYC* gene locus. However, because the distal enhancers are juxtaposed to sequences upstream and around the *MYC* transcription start site, perhaps a more detailed analysis of Wnt responsive elements within the proximal promoter is warranted. Although delimiting the precise elements at the *MYC* promoter that control Wnt/ß-catenin-dependent activation of *MYC* expression in colon cancer cells is a daunting task, it will ultimately be required to understand the relationship between de-regulated Wnt signaling and colon cancer pathogenesis.

## Supporting Information

Figure S1 **MYC expression levels are higher in HCT116 colon cancer cells relative to levels in HEK293 cells.** (**A**) RNAs were isolated from HCT116 and HEK293 cells and cDNAs were synthesized using reverse transcriptase. Real-time PCR with primers specific to the *MYC* gene was used to detect *MYC* expression. Error bars represent SEM. (**B**) Proteins isolated from each cell type were separated by SDS-PAGE and analyzed by western blot using antibodies specific for MYC or ß-tubulin. (**C**) Relative MYC protein levels in HCT116 and HEK293 cells. MYC and ß-tubulin bands in **B** were quantified using ImageJ software.
(EPS)

Table S1 PCR primer sequences, chromosomal positions of ß-catenin ChIP-Seq peaks and the positions of consensus TCF4 motifs within the ß-catenin bound peak regions.
(XLSX)
